# Effect of obturation techniques on fracture resistance of endodontically treated teeth

**DOI:** 10.6026/973206300210361

**Published:** 2025-03-31

**Authors:** Divya Batra, Ankita Dixit, Jasmine Marwaha, Niladri Maiti, Tejal Gowda, Ayan Saha

**Affiliations:** 1 Department of Conservative Dentistry and Endodontics, National Dental College and Hospital, Derabassi-140507, India; 2 Department of Pedodontics and Preventive Dentistry, Government Dental College and Hospital, Jamnagar, Gujarat-36100, India; 3 Department of Conservative Dentistry and Endodontics, Maharishi Markandeshwar College of Dental Sciences and Research, Mullana-133203, Haryana, India; 4 Department of Dentistry, Central Asian University, Uzbekistan; 5 Department of Conservative Dentistry and Endodontics, Dayanand Sagar College of Dental Sciences, Karnataka, India; 6 Department of Dentistry, Kalinga Institute of Dental Sciences, Bhubaneswar, Odisha, India

**Keywords:** Obturation techniques, fracture resistance, endodontically treated teeth, cold lateral compaction, warm vertical compaction, single cone technique, bioceramic sealer

## Abstract

The success of endodontic treatment depends on the obturation method that influences the structural strength of treated teeth. Therefore, it is of interest to evaluate the fracture resistance of 60 single-rooted premolars using Cold Lateral Compaction, Warm Vertical Compaction, or the Single Cone Technique with Bioceramic Sealer, followed by compressive testing after four weeks in artificial saliva. The Single Cone Technique with Bioceramic Sealer showed the highest fracture resistance (600±55 N) followed by Warm Vertical Compaction (520±45 N), while Cold Lateral Compaction had the lowest resistance (450±50 N) (p<0.05). This shows that the Single Cone Technique with Bioceramic Sealer provides superior reinforcement, whereas Cold Lateral Compaction is least effective. Thus, clinicians should consider obturation techniques based on their impact on tooth strength to improve long-term treatment outcomes.

## Background:

The main purpose of endodontic treatment comes from its process to eliminate infections while safeguarding natural teeth and restoring their operational capacity. The structural weakness resulting from dentin removal combined with dehydration and instrumentation extends root-treated teeth to fracture susceptibility [1, 2]. The method used for obturation alongside materials play a decisive role in tooth fracture resistance by supplying additional support to tooth structures and preventing voids that reduce strength [3]. Current clinical practice uses three major obturation technologies which include Cold Lateral Compaction and Warm Vertical Compaction together with Single Cone Technique utilizing Bioceramic Sealer. The Cold Lateral Compaction technique is frequently applied in clinics because it combines ease of use and economic effectiveness yet struggles to achieve proper canal wall fit thus generating areas of weakness throughout the root structure [4, 5]. The procedure of Warm Vertical Compaction enables improved canal adaptation and produces more uniform fill material which enhances root fracture resistance [6]. The Single Cone Technique with bioceramic sealers is gaining popularity because it maximizes root canal wall bonding potential as well as root wall strengthening capacity [7, 8]. Research findings about endodontically treated teeth fracture resistance following different obturation methods remain inconsistent according to various study results. Research findings continue to be divided about whether thermo plasticized obturation procedures improve resistance to fracture or if sealer selection with proper dentin interaction proves more impactful [9, 10].

Endodontically treated tooth structure depends on both the filling technique alongside the selected sealer material. Clinicians have widely used traditional sealers which incorporate zinc oxide eugenol and epoxy resin-based products because they demonstrate strong sealing properties and antimicrobial activities. The new class of bioceramic-based sealers combines excellent bonding performance with biological activity while simultaneously showing potential to strengthen the root canal walls [11]. The chemical connection between dentin and root canal filling material at the wall interface created by these sealer materials boosts their adhesiveness while decreasing microleakage and structural failure incidents [12]. The formation of hydroxyapatite at sealer-dentin interfaces by bioceramic sealers works to strengthen the root according to research [13]. Two key external factors along with occlusal forces determine the lifespan of root canal-treated teeth together with remaining coronal tooth structure and post-endodontic restorations. Internal root support during post-treatment success depends on proper root filling technique because it distributes force evenly while stopping crack growth in the walls [14]. Proper implementation of the Warm Vertical Compaction method distributes forces evenly through the root canal system thereby decreasing the risk of vertical root fractures according to literature review [15]. By integrating knitted single cones with bioceramic sealers the technique gains properties of both expansion and bone-bonding which offset the lack of compaction to preserve structural integrity [6]. Therefore, it is of interest to compare different obturation techniques on endodontically treated tooth fracture resistance to find the most efficient method for long-term clinical success.

## Materials and Methods:

Sixty human premolars with single canals were used in this study after extraction. The study omitted any teeth with fracture lines, resorption defects, endodontic history and cracks. Standardized root lengths of 14 mm occurred after the teeth received cleaning processes followed by storage in 0.9% saline solution. The rotary nickel-titanium instrumentation system prepared all teeth until reaching the #40 apical sizes with 0.06 taper. The clinician's utilized 2.5% sodium hypochlorite solution during irrigation followed by 17% EDTA and distilled water irrigation of the canals before the completion of the procedure. Consolidation of the canals took place using sterile paper points. The researchers distributed the teeth into three separate groups (N=20) according to which obturation method was employed. The first process involved fitting resin-based sealer onto working length Gutta-percha cones before applying lateral cold compaction in this group.

The accessory cones received spreader pressure until the entire canal space became filled. The second group used a master gutta-percha cone coated with resin-based sealer which was placed initially and then followed by successive heated softened gutta-percha increments compacted using a heated plugger. In Group 3 investigators used a single gutta-percha cone to reach working length while they applied the bioceramic sealer to both canal walls and refrained from lateral and vertical condensation. The researchers placed all specimens into artificial saliva at 37°C after obturation until the four-week simulation of intraoral duration was complete. Acrylic blocks received the roots with two millimeters of the coronal segment exposed for testing. Testing equipment utilized a universal testing machine to measure fracture resistance by applying a compressive force with a speed of 1 mm/min until obtaining tooth fracture. The recording device measured the breakage force of every test specimen as Newtons (N). One-way analysis of variance served to evaluate and determine any statistical differences among the fracture resistance data from the three experimental groups. Tukey's test followed the analysis to derive the results of pairwise group comparisons. Statistical significance was set at p<0.05.

## Results:

The fracture resistance assessment values of the three obturation methods differed substantially between groups. The Single Cone Technique using Bioceramic Sealer produced the greatest mean resistance to fracture while the Warm Vertical Compaction technique came in second position with lowest values attending to the Cold Lateral Compaction technique. The Table 1 shows both mean fracture resistance values and standard deviations (SD) for each tested group. Bioceramic Sealer using the Single Cone Technique attained a mean resistance of 600 ± 55 N which proved to be more resistant than the 520 ± 45 N obtained with Warm Vertical Compaction and the 450 ± 50 N resistance from Cold Lateral Compaction. ANOVA results demonstrated significant differences between the studied groups based on p<0.05. The Single Cone Technique with Bioceramic Sealer surpassed both the Warm Vertical Compaction and the Cold Lateral Compaction techniques regarding fracture resistance according to Tukey's test analysis (p<0.05) and exhibited higher resistance than the other two methods (p<0.05). The findings indicate that the choice of obturation technique significantly affects the fracture resistance of endodontically treated teeth (Table 1). The use of bioceramic sealers in the Single Cone Technique appears to reinforce the root structure, contributing to higher fracture resistance.

## Discussion:

Results from this investigation demonstrate that different endodontic filling procedures remarkably affect endodontically treated teeth resistance to fracture. Endodontically treated teeth obtained their highest fracture resistance when using the Single Cone Technique with Bioceramic Sealer but performed second best with Warm Vertical Compaction and showed the lowest resistance when treated with Cold Lateral Compaction. Obturation procedures have been confirmed to directly influence the structural stability of the root canal during treatment. The lower fracture resistance in Cold Lateral Compaction obturated canals mostly results from creation of voids and gaps because of the method. The lateral placement of accessory cones in this method struggles to achieve proper sealing and stress uniformity because it weakens the root structure [1, 2]. The spreader produces mechanical wedging that creates tension in dentinal structures adjacent to the canal which raises their risk of fracturing [3, 4]. The Warm Vertical Compaction method achieved improved fracture resistance because it provided better gutta-percha adaptation to canal walls together with decreased void formation [5, 6]. Thermoplastic capabilities of gutta-percha during this method permit it to adopt canal shapes better while distributing stress evenly [7]. Research findings show that warm obturation approaches strengthen the dental fractures by producing uniform obturation material consolidation and lowering canal seepage [8, 9].

When using the Single Cone Technique with Bioceramic Sealer the highest level of fracture resistance was noted possibly because bioceramic sealers offer exceptional dentin-binding capabilities and chemical bonding abilities to dentin [10, 11]. Bioactive properties in these sealers help bond with dentin tissue better while strengthening root canal structures because they create hydroxyapatite crystals near the sealer-dentin boundary [12]. Bioceramic sealers maintain excellent sealing properties through their stable dimensions alongside reduced polymerization shrinkage thus reinforcing the root structure [13, 14]. Previous investigations confirm bioceramic sealer performance exceeds that of conventional resin and zinc oxide-eugenol sealer types regarding fracture resistance [15]. The suggested research method provided important findings yet researchers must recognize some key restrictions. Because the experiment took place outside a living body researchers cannot replicate all effects that occur within the oral cavity including tooth resistance changes due to dynamic forces alongside changes in temperature and humidity. The study examined single-rooted premolars exclusively but researchers require further investigation of obturation effects on teeth with multiple roots together with various canal arrangements. The choice of sealer and obturation technique plays a crucial role in enhancing the fracture resistance of endodontically treated teeth so also the obturation technique has an insignificant effect [16, 17-18].

## Conclusion:

Proper obturation methods becomes essential for improving endodontically treated tooth resistance against fracture. The Single Cone Technique which uses Bioceramic Sealer delivers optimal results concerning root reinforcement and stress distribution. However, the long-term behaviour of these filling methods when loaded under clinical conditions needs to be investigated.

## Figures and Tables

**Table 1:** Fracture resistance (MEAN ± SD) of endodontically treated teeth obtained using different obturation techniques

**Group**	**Obturation Technique**	**Mean Fracture Resistance (N) ± SD**
1	Cold Lateral Compaction	450 ± 50
2	Warm Vertical Compaction	520 ± 45
3	Single Cone with Bioceramic Sealer	600 ± 55
